# Drip Loss During Strawberry Thawing: A Simple Method for Predicting and Improving the Colour Stability of Nectars

**DOI:** 10.1155/ijfo/6657122

**Published:** 2026-04-02

**Authors:** Helen Murray, Jhoan-Sebastian Rincon-Berbeo, Kristýna Šimková, Marlene Lindner, Carine Le Bourvellec, Jerneja Jakopič, Heidrun Halbwirth, Manfred Gössinger

**Affiliations:** ^1^ Department of Fruit Processing, Federal College and Institute for Viticulture and Pomology, Klosterneuburg, Austria; ^2^ INRAE, Avignon University, Avignon, France; ^3^ Department of Agronomy, University of Ljubljana, Ljubljana, Slovenia, uni-lj.si; ^4^ Institute of Chemical, Environmental and Bioscience Engineering, Vienna University of Technology, Vienna, Austria, tuwien.ac.at

**Keywords:** anthocyanin, colour acceptance, defrosting, shelf-life, strawberry puree

## Abstract

Drip loss is the liquid released from frozen strawberries during defrosting. High levels of drip loss make strawberries unsuitable for many applications that require whole fruit. This study is aimed at investigating the relationship between strawberry drip loss and the colour stability of nectars produced from these strawberries. Both solid (remainder after drip removal during defrosting) and liquid fractions (drip loss) were analysed by HPLC for anthocyanin, sugar and acid concentration, as well as total soluble solids and titratable acidity, to compare differences between the fractions. The results showed a strong correlation between strawberry drip loss and the colour stability of the resulting nectars. Strawberries with high levels of drip loss produced nectars with more stable colour. Determining the percentage of drip loss provides a quick and low‐cost method for industrial nectar producers to select strawberries suitable for the production of colour‐stable nectars. Furthermore, it was demonstrated for the first time that higher levels of solid content, achieved by separating the drip loss of defrosted strawberries before processing into puree, lead to nectars with higher initial consumer acceptance factor (AF) and a higher AF after 12 weeks. As a result, these nectars had a significantly better colour stability.

## 1. Introduction

Drip loss refers to the amount of liquid released from frozen fruits and vegetables when they are thawed [1]. It occurs as a result of ice crystals forming within the fruit during freezing, which damage the cell walls, altering the cell structure and osmotic pressure within the cell [2]. This damage diminishes the water‐holding capacity of the fruit, causing liquid to be released during thawing [3]. In the strawberry (*Fragaria x ananassa*) industry, drip loss presents significant challenges for commercial processors, as excessive drip loss compromises the shape and texture of the fruit, rendering it unsuitable for many applications [4, 5].

Although freezing and thawing reduce product quality [6–9], freezing remains a vital preservation technique, given the short shelf‐life of strawberries [10]. Minimising drip loss has long been an objective within the industry, with numerous studies aimed at identifying cultivars that produce the least amount of drip [4, 5, 11, 12] as well as developing prefreezing treatments to reduce drip loss [13–16]. Drip loss in strawberries is strongly affected by freezing conditions. Research has shown that strawberries frozen slowly, stored in frozen conditions for prolonged periods, and subjected to fluctuating storage temperatures release the highest quantity of drip [17, 18]. A negative correlation has been observed between firmness and drip loss [4, 19], with softer fruits tending to release more liquid. Strawberry firmness declines during ripening [20–22], and nectars produced from riper fruits have better colour stability [23]; therefore, softer fruits result in nectars with improved colour stability [21, 24]. Despite the extensive previous research on strawberry drip loss, and these established links between drip loss, firmness and colour stability, drip loss has not previously been investigated for its link with colour stability of strawberry products during storage.

Good colour stability in strawberry nectars is important, as consumers are unlikely to purchase nectars with degraded colour. Consumer acceptance of the colour of a strawberry can be quantified by an acceptance factor (AF). The AF is calculated from CIELAB colour coordinates and reflects consumer acceptance of the colour of strawberry nectars by correlating with the likelihood of a consumer deeming the colour acceptable. An *A* *F* < 0.4 corresponds with a nectar that is unacceptable to consumers as it has undergone excessive browning, whereas an *A* *F* > 0.7 indicates excellent colour acceptance. [25]. Techniques for predicting colour stability have been developed [21, 26, 27], but these typically require specialised instrumentation that may not be practical or accessible for small‐scale producers. In comparison, drip loss can be quantified using simple methods, such as suspending strawberries (e.g., in a funnel) and weighing the collected liquid to calculate the percentage of fruit weight lost [17, 28].

This study investigated whether the percentage of drip loss from thawed strawberries correlates with, and can subsequently predict, the colour stability of nectars made from these strawberries. Additionally, this research examined the differences between the solid and liquid fractions of defrosted strawberries, with particular emphasis on variations in the profile and concentration of anthocyanin components between the fractions. This focus arises from evidence that higher anthocyanin levels improve the colour stability of strawberry products [21, 27, 29, 30]. However, other studies concluded that either the concentration alone did not have an impact [23], or that the anthocyanin profile—rather than its concentration—dictates the stability [31]. Therefore, a closer examination of this issue is necessary, as previous studies have focused on minimising drip, rather than identifying the differences between the fractions.

This study also evaluated the impact on colour stability when either the liquid or solid fraction is partially lost. This is important as during both industrial processing and scientific studies there is a risk of inadvertently lost—such as during sieving (where solids may be lost) or during thawing (where liquid may be lost, for example if containers are not watertight). The impact of removing or altering the amount of liquid lost has not previously been investigated and could have large implications for colour stability. To this end, nectars were produced with altered solid and liquid compositions.

## 2. Material and Methods

### 2.1. Strawberries

#### 2.1.1. Strawberries for Drip Loss Calculation

During June and July 2021, a total of 63 variants of strawberries (5‐kg samples) were harvested for use in nectar production. These included 47 variants—representing 14 different cultivars, gathered at various stages of ripeness (ripe [red skin] and overripe [dark red skin]) and from different harvest dates (where possible each cultivar was harvested twice)—sourced from two locations in Lower Austria (Zeiselmauer [13 cultivars–43 variants] and Raasdorf [2 cultivars–4 variants]). One cultivar (Malling Centenary) was obtained from both locations, with a third harvest from a separate field in Raasdorf. From Zeiselmauer, eight cultivars were harvested ripe and overripe twice in the season, to compare the effects of ripening and harvest point. An additional 16 variants of strawberries—comprising five cultivars, also picked at varying ripeness levels (ripe and overripe for all cultivars) and harvest points (two cultivars—‘Honeoye’ and ‘Elsanta’—harvested three times in the season)—were obtained from Poland. The strawberries were harvested in the morning, packed into 5‐kg vacuum‐packed (Austrian samples) or ziploc (Polish samples) plastic bags, which were both permeable to oxygen. They were frozen by placing into a forced air fan–assisted walk‐in freezer at −18°C within 2 h of harvesting. The rate of freezing from ambient to 0°C was 2.4°C/h, and the rate of cooling from 0°C to −18°C was 0.3°C/h. During storage, temperature fluctuations were kept to a minimum, although the opening and closing of the doors gave fluctuations of ± 2°C.

#### 2.1.2. Strawberries for Solid–Liquid Separation Tests

The cultivar ‘Honeoye’ was chosen to assess the stability of nectars produced from purees containing varying proportions of liquid and solid fractions following thawing. ‘Honeoye’ was selected from the samples harvested from Poland, as it is currently a widely used and commercially important industrial cultivar, and therefore was available in large quantities. In addition, there was a large proportion of drip in these fruits, which allowed for the necessary quantities for producing the wide range of percentages. Three strawberry variants were utilised: overripe strawberries harvested on 24 June 2021, and ripe and overripe strawberries harvested on 30 June 2021.

### 2.2. Methods

#### 2.2.1. Preparation of Nectars

##### 2.2.1.1. Defrosting of Strawberries

For the nectars of the 67 variants of strawberries, frozen strawberries were thawed for 24 h at 20°C, by placing a 5‐kg plastic bag in a plastic bucket. The drip and berries remained together in the bag before processing.

For the ‘Honeoye’ strawberries used to produce nectars with different solid contents, strawberries were placed in large funnels (15 kg per variant, divided into three funnels, with 5 kg per funnel), with containers positioned underneath to collect the drip. The strawberries were left to defrost and separate for 24 h at 20°C. After this time, the liquid fraction had completely fallen into the containers, whereas the solid fraction remained in the funnels. The solids and liquids were kept separate.

##### 2.2.1.2. Puree and Nectar Preparation

Purees were obtained by processing with a rotor mill, equipped with a 1‐mm sieve (Feuma, Gößnitz, Germany). Strawberries that did not have their drip separated were placed (5 kg) into a hopper on top of the rotor mill, together with their drip, and were allowed to enter the rotor as the puree processed through the sieve. Strawberries were processed at the top speed until no more puree was obtained.

For the ‘Honeoye’ samples that had their drip removed, the solid fraction (the remains from the funnel) were processed through the rotor mill on top speed until no further puree was obtained, whereas the liquid drip was kept separate. Solids and liquids were reconstituted into purees, by adding the liquid drip back to the pureed solid fraction in different proportions. The proportions were *w*/*w* of processed solids and liquid drip. The reconstituted purees were made with 0% (i.e., only drip), 5%, 25%, 50%, 75% and 100% (i.e., only processed solids) solids. Purees were reconstituted in the morning, kept in lidded plastic 5‐kg buckets, and the buckets placed into a walk‐in refrigerator at 4°C until nectar processing. Nectars were produced from the 5%, 10%, 50% and 75% samples on the day of puree production. Due to time and equipment constraints, purees with 0%, 25% and 100% were kept at 4°C for circa 18 h and processed into nectar the following day (circa. 18 h). The order of nectar production was selected to allow the maximum processing, by pairing nectars for pasteurisation that were suitably different in appearance, and ensuring all variants were processed in the same order. Additionally, as it was an aim to directly compare the solid and liquid fractions, so it was ensured that these were kept at 4°C for the same amount of time to ensure full comparison, as storage at 4°C can lead to degradation of anthocyanin pigments. This is due to enzymes (PPO/POD) that are inactive and physically separated in the cell during frozen storage, but after thawing and pureeing they come into contact with phenolic compounds, which they then degrade [6, 7].

Nectars were prepared from puree by a method described in Murray et al. [27]. Purees were blended with water, citric acid and sugar to formulate nectar comprising 40% puree, 15°Brix and 7.0 g/L titratable acidity (TA), and then homogenised using a hand blender (Philips, Drachten, Netherlands) until all solids were dissolved and well mixed. A quantity of 140 ± 1 *g* of nectar was dispensed into glass jars (212 mL), sealed with screw‐top lids, and pasteurised in a waterbath (Westfalia, Hagen, Germany) at 80°C for 10 min. The finished nectars were stored in the dark at 20^°^ *C* ± 3^°^ *C*.

#### 2.2.2. Physical and Chemical Analysis

##### 2.2.2.1. Total Soluble Solids (TSS), pH Values and TA

TSS (°Brix), pH values and TA were measured according to the method described in Murray et al. and the method description partly reproduces the wording [27]. TSS was evaluated using a hand‐held refractometer (N‐20, Brix range: 0%–20%, ATAGO, Tokyo, Japan). TA was determined by titration to a pH end point of 7.0 (using 0.1 N NaOH Titrisol, Merck KGaA, Darmstadt, Germany) and the result multiplied by the tartaric acid factor (0.75) to express acidity as g/L. pH measurements were conducted with a pH meter (MultilineP4, WTW, Weilheim, Germany) equipped with a pH electrode (SenTix 41‐3, WTW, Weilheim, Germany).

##### 2.2.2.2. Drip Loss

Drip loss was calculated by placing a known weight of frozen strawberries (frozen for > 6 months at −18°C) into a funnel positioned above a measuring cylinder. The fruit was left for 24 h at ambient temperature (20°C–25°C) and allowed to drip into the cylinder. Drip loss experiments were carried out in duplicate. The weight of the drip was calculated, and drip loss was expressed as a percentage (Equation 1):

(1) Drip loss %=weight of drip/initial weight of strawberries×100.

##### 2.2.2.3. Firmness

Firmness measurements were obtained from the Austrian strawberry samples using a penetrometer (Mecmesin, AFG 500N, stamp 11.55 mm, United Kingdom), following the method described in Murray et al. [21]. The speed of penetration was 1 cm/s, and the firmness was expressed in kg/cm^2^. From each strawberry variant, 10 fresh strawberries were measured on two sides of each fruit, resulting in a total of 20 measurements per variant.

#### 2.2.3. Colour Measurements

As reported in Murray et. al [27], colour measurements (*L* ∗—lightness, *a* ∗—green (negative) to red, and *b* ∗ blue (negative) to yellow) were conducted using a CM‐5 spectrophotometer (Minolta, Osaka, Japan). Nectars were measured in a 30‐mm petri dish on reflectance mode. Clear juices were measured in transmission mode, using a 10‐mm cell. Chroma (*C* ∗—colour saturation or intensity) and hue angle (*h°*—colour hue on a wheel described in degrees) were calculated according to previously published methods [32]. The AF was determined using the formula *A* *F* = *a*∗/*h* as outlined by Gössinger et al. [25]. Nectars were assessed for colour following pasteurisation on day of production (as described in Section 2.2.1.3) and subsequently on a weekly basis over a 12‐week period. Two samples of each nectar were measured in duplicate, to give four measurements per nectar. Following Murray et al. [27], colour stability was evaluated using several indicators: AF4, AF8 and AF12 represent the AF values at Weeks 4, 8 and 12, respectively. D4, D8 and D12 indicate the change in AF between the day of production and Weeks 4, 8 and 12, respectively.

#### 2.2.4. Anthocyanin Determination

##### 2.2.4.1. Extraction of Phenolic Compounds

Polyphenol extracts were obtained directly from freeze‐dried nectar through a crude extraction method, utilising the procedure outlined in Brahem et al. [33] with some modifications. A total of 25 mg of lyophilised samples were dissolved in 1500 *μ*L of dried methanol acidified with acetic acid (10 mL/L). The mixtures were shaken at 1500 rpm for 10 min using an MS‐100 Thermo Shaker (Allsheng, Hangzhou, China), followed by centrifugation (Hettich Universal 320 R centrifuge, Tuttlingen, Germany) at 9000 rpm and 4°C for 5 min. The resulting supernatant was collected, and the remaining residue was reextracted under identical conditions. The combined supernatants were then concentrated by evaporating the solvent under a nitrogen stream at 34°C, reducing the volume to 1500 *μ*L. Extracts were stored at −20°C until further analysis. All extractions were performed in triplicate.

##### 2.2.4.2. Identification of Phenolic Compounds by HPLC/ESI‐MS^2^

HPLC/ESI‐MS2 analysis was conducted using an Acquity Ultra performance LC (UPLC) system (Waters, Milford, Massachusetts, United States), equipped with a photodiode array detector (monitoring at 280, 320, 350 and 520 nm), coupled to a Bruker Daltonics (Bremen, Germany) HCT ultra ion trap mass spectrometer with an electrospray ionisation (ESI) source. Chromatographic separation was achieved using a Luna omega 3‐*μ*m polar C‐18 100‐Å column (Phenomenex, Torrance, California, United States) maintained at 30°C. The mobile phase was composed of water/formic acid (98:2, *v*/*v*) (eluent A) and acetonitrile (eluent B), with a flow rate of 1 mL/min. The gradient elution programme was as follows: 3%–9% B (0–5 min); 9%–16% B (5–15 min); 16%–50% B (15–45 min); 50%–90% B (45–48 min); and 90% B maintained from 48–52 min. An injection volume of 5 *μ*L was used. Mass spectrometric detection was carried out in both negative ion mode (for phenolic compounds) and positive ion mode (for anthocyanins), covering a mass range of *m*/*z* 100–1200. The MS parameters for both ionisation modes were as follows: capillary voltage ± 2 kV, nitrogen gas flow at 8 L/min, desolvation temperature at 365°C and nebuliser pressure at 50 psi. Data acquisition and processing were performed using Compass and DataAnalysis 4.3 software (Bruker Daltonics, Bremen, Germany) following the approach in Tarone et al. [34].

##### 2.2.4.3. Quantification of Polyphenols by HPLC‐DAD

Phenolic compounds were quantified using reversed‐phase high‐performance liquid chromatography coupled with diode array detection (RP‐HPLC‐DAD) (Shimadzu, Kyoto, Japan). The system included a SIL‐20ACHT Prominence autosampler, two pumps, LC‐20AD Prominence liquid chromatograph UFLC, a DGU‐20A5 Prominence degasser, a CTO‐20AC Prominence column oven, a SPD‐M20A Prominence diode array detector, and a CBM‐20A Prominence communication bus module, operated by a LC Solution software. Chromatographic separations were carried out on a Luna omega 3‐*μ*m polar C‐18 100‐Å column (Phenomenex, Torrance, California, United States) maintained at 30°C. The mobile phase consisted of water/formic acid (98:2, *v*/*v*) (eluent A) and acetonitrile (eluent B). The flow rate was set at 1 mL/min. The gradient elution programme was as follows: 3%–9% B (0–5 min); 9%–16% B (5–15 min); 16%–50% B (15–45 min); 50%–90% B (45–48 min) and 90% B held from 48–52 min. Crude extracts were injected at a volume of 20 *μ*L. Individual phenolic compounds were quantified in mg/kg of fresh weight (FW) by external calibration with reference standards, detected at specific wavelengths: 280 nm for (+)‐catechin, (−)‐epicatechin; 320 nm for phenolic acid derivates; 350 nm for flavonols and 520 nm for cyanidin and pelargonidin glycosides.

#### 2.2.5. Sugars and Organic Acids Extraction and Determination

The extraction of organic acids and sugars was performed using 1 g of puree combined with 5 mL of bidistilled water. Following the method described by Simkova et al. [35], samples were shaken for 30 min, and after extraction centrifuged for 10 min at 10,000 × *g* at 4°C (Eppendorf Centrifuge 5810 R; Hamburg, Germany). The supernatant was subsequently filtered using 0.20 *μ*m cellulose filters (Macherey‐Nagel, Düren, Germany). Organic acids were analysed using the Vanquish HPLC system (ThermoScientific, Waltham, Massachusetts, United States) equipped with a Rezex ROA‐Organic acid *H* + 8*%* column (150 *x* 7.8 *m* *m*; Phenomenex, Torrance, California, United States). The mobile phase consisted of 4‐mM sulphuric acid in bidistilled water. Temperature was 65°C, the flow rate was 0.6 mL/min and injection volume was 20 *μ*L, detection of sample response was carried out at 210 nm using a UV detector. Organic acids were identified using external standards for citric, malic and fumaric acids from Fluka Chemie (Buchs, Switzerland) and shikimic acid from Sigma‐Aldrich (Steinheim, Germany).

Individual sugars were analysed by the Vanquish HPLC system (ThermoScientific, Waltham, Massachusetts, United States) using a Rezex RCM‐monosaccharide *C* *a* + 2*%* column (300 *x* 7.8 *m* *m*; Phenomenex, Torrance, California, United States), with bidistilled water as the mobile phase. Temperature was 65°C, the flow rate was 0.6 mL/min and injection volume was 20 *μ*L; individual sugars were identified using external standards for fructose, glucose and sucrose (Fluka Chemie GmBH, Buchs, Switzerland). All other parameters for HPLC analysis and the quantification of sugars and organic acids were applied as described by Simkova et al. [35].

#### 2.2.6. Ascorbic Acid Extraction and Determination

Ascorbic acid extraction was performed using 2.5 g of puree and 2.5 mL of 3% metaphosphoric acid. Following the procedure described in Simkova et al. [35], samples were shaken for 30 min at room temperature. Following extraction, the mixture was centrifuged for 10 min at 7000 × *g* at 4°C (Eppendorf Centrifuge 5810 R; Hamburg, Germany). The resulting supernatant was subsequently filtered through 0.20‐*μ*m cellulose filters (Macherey‐Nagel, Düren, Germany). The ascorbic acid content was analysed using a Vanquish HPLC system (ThermoScientific, Waltham, Massachusetts, United States) equipped with a Rezex ROA‐Organic acid *H* + 8*%* column (150 *x* 7.8 *m* *m*; Phenomenex, Torrance, California, United States). The mobile phase consisted of 4‐mM sulphuric acid in bidistilled water. Temperature was 20°C, the flow rate was 0.6 mL/min and the injection volume was 20 *μ*L as described in Simkova et al. [35]. Sample response was measured with a UV detector at 245 nm and ascorbic acid was identified using an external standard from Sigma‐Aldrich (Steinheim, Germany).

#### 2.2.7. Statistical Analysis

Statistical analyses were performed using IBM SPSS 29 version: 29.0.0.0 (241) (Statistical Package for the Social Sciences) (IBM Corp, Armonk, New York, United States). Analysis of variance (ANOVA) followed by post hoc Tukey‐HSD was performed to determine significant differences (*p* < 0.05) between the different percentages of solid content and their AF0, AF12 and D12 (four nectar measurements per variant, three variants per percentage for a total of 12 included values), and their TSS and TA (1 value per variant, 3 values total). ANOVA and Tukey‐HSD were also used to determine significant differences in drip loss between different countries (Austria: 48 variants and Poland: 15 variants), cultivars, ripening stages and harvest points. Independent *t*‐tests were used to find significant differences (*p* < 0.05) between the liquid drip and solid fraction for their anthocyanin, sugar and acid content (3 values per variant, 9 values per variant). Pearson correlation coefficients were performed to determine significant (*p* < 0.05) correlations between the means of the AF0 (4 values per variant), AF12 (4 values per variant), D12 (4 values per variant), firmness (10 values per variant) and drip loss % (2 values per variant). Simple linear regression was used to determine if firmness and drip loss could be used to significantly (*p* < 0.05) predict AF12 and D12.

## 3. Results and Discussion

### 3.1. Drip Loss

#### 3.1.1. Drip Loss as a Predictor of Colour Stability

Figure 1 presents the relationship between the average percentage of drip loss of each strawberry variant and the average values of AF of nectars produced from these strawberries, on day of nectar production, after pasteurisation (AF0), after 12 weeks of storage (AF12) and the difference between these two time points (D12). A weak but statistically significant correlation was observed between the average drip loss percentage and AF0 (*r* = 0.360, *p* = 0.004). The correlation with AF12 was notably stronger (*r* = 0.669, *p* < 0.001). Consequently, a significant negative correlation was found between drip loss and D12 (*r* = −0.605, *p* < 0.001). Simple linear regression was employed to assess whether the percentage of drip loss significantly predicted AF12 and D12 values.

Figure 1The mean strawberry weight lost in liquid in percentage of starting weight when defrosted at room temperature for 24 h, plotted against the mean acceptance factor of nectars made from these strawberries (a) after initial production (AF0), (b) after 12 weeks of storage at 20°C (AF12), and (c) the difference between these two measurements (D12).

**Figure:**
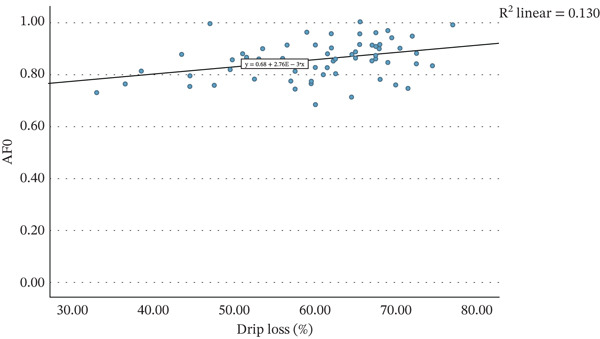
(a)

**Figure:**
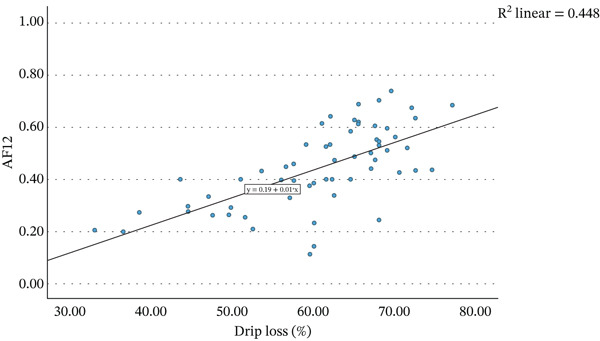
(b)

**Figure:**
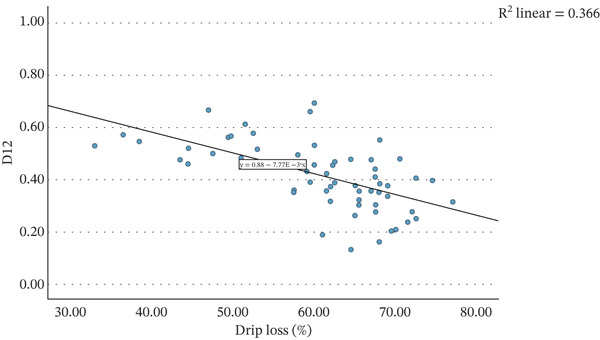
(c)

Figure 1b displays the regression model describing the relationship between drip loss percentage and AF12. The regression was statistically significant (*R* ^2^ = 0.448, *F*(1, 61) = 49.4, *p* < 0.001) indicating that 44.8% of the variability in AF12 could be explained by drip loss. The regression coefficient (*B* = 0.010, 95% CI [0.007, 0.013]) suggests that an increase of 1 percentage point in drip loss corresponds to an increase in AF12 by 0.010. This is very small, as an increase of 10 percentage points in drip loss would only increase the AF12 by 0.1, although 0.1 difference in AF does reflect a marked change in acceptability. Additionally, the predictive ability is only moderate, and so relying on drip loss alone to predict colour stability could lead to extensive inaccuracies. Nevertheless, drip loss could still screen raw material to determine raw material that was more suitable for nectar production (high drip loss) than those more suitable for whole defrosted strawberries.

Figure 1c depicts the regression model for the relationship between drip loss and D12. This regression was also statistically significant (*R* ^2^ = 0.366, *F*(1, 61) = 35.2, *p* < 0.001), although was weaker than the model for AF12, with drip loss explaining 36.6% of the variance in D12. The regression coefficient (*B* = −0.008, 95% CI [−0.005, −0.010]) indicates that for each 1 percentage point increase in drip loss, D12 decreases by 0.008.

The observed relationship between colour stability and drip loss suggests that quantifying drip loss could provide a rough, simple and practical approach to identify strawberries best suited for nectar production. This method would be particularly valuable for small‐scale processors, as it does not require the special equipment or technical expertise necessary for using other, more accurate, prediction models [21, 26, 27], but could instead give a guide for producers that would otherwise be unable to screen material. However, there are limitations to this model, as it is advisable to compare only strawberries that have been frozen using the same method and stored for similar durations, since variations in freezing conditions can significantly impact drip loss [17, 18]. In industrial settings, such effects are generally minimised due to standardised freezing procedures. Additionally, the relatively small effect that drip loss has, and the effect that drip loss is only responsible for 44% of the variance in the AF12 means that relying on drip loss alone could lead to false positives and negatives. Despite these restrictions, employing drip loss could still be of use as a screening tool that could aid in determining which strawberries are optimal for nectar production and which are better suited for freezing whole, as low‐drip strawberries are preferred for their ability to retain structure after thawing [5].

#### 3.1.2. Drip Loss Variation With Country, Cultivar, Ripeness and Harvest Point

**Figure 2:**
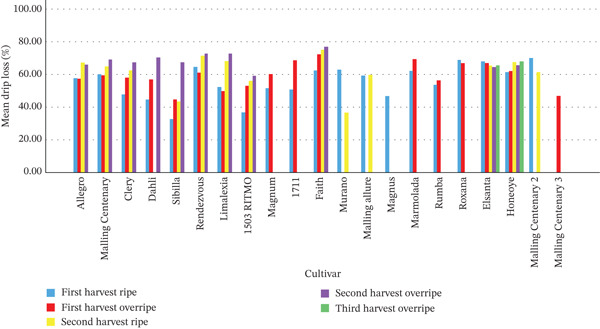
Mean drip loss in percentage of each sample, defined by cultivar and relative harvest and ripeness point; where the same cultivars from multiple locations were obtained (‘Malling Centenary’) these are labelled numerically.

As shown in Figure 2, drip loss was found to vary with cultivar, ripening stage, harvest point and country of origin. Strawberries from Poland (*m* *e* *a* *n* = 64.54*%* ± 4.69*%*) had significantly higher levels of drip than samples from Austria (*m* *e* *a* *n* = 59.05*%* ± 10.85*%*), (*t*(124) = −2.77), *p* = 0.007. This is possibly explained by the fact that in Poland strawberries are cultivated specifically for processing, and are harvested at a more ripe point, which is known to result in nectars with lower firmness and higher colour stability [24]. This is supported by the overripe strawberries in this study (*m* *e* *a* *n* = 63.16*%* ± 7.98*%*) having significantly higher levels of drip than the ripe strawberries (*m* *e* *a* *n* = 57.97*%* ± 10.91*%*, (*t*(124) = −3.02), *p* = 0.003. When considering only the cultivars where two harvests of both ripe and overripe (four variants per cultivar) strawberries were obtained (‘Sibilla’, ‘1503 Ritmo’, ‘Clery’, ‘Limalexia’, ‘Allegro’, ‘Malling Centenary’, ‘Honeoye’, ‘Elsanta’, ‘Rendezvous’ and ‘Faith’), the first ripe harvest had significantly (*p* < 0.05) lower amounts of drip (54.36*%* ± 12.06*%*) than overripe strawberries from the second harvest (68.07*%* ± 5.08*%*). But there were no significant (*p* < 0.05) differences between the first harvest ripe and first harvest overripe (58.47*%* ± 8.09*%*), first harvest overripe and second harvest ripe (64.13*%* ± 8.68*%*), and second harvest ripe and second harvest overripe. When averaged across these four harvest and ripeness points, ‘Faith’ was the cultivar with the highest amount of drip (71.55*%* ± 5.83*%*), whereas ‘Sibilla’ had the lowest quantity (47.03*%* ± 13.73*%*). This shows that the drip loss of a strawberry varies by its cultivar, ripeness stage and harvest point. This is in agreement with previous studies that found that different cultivars of strawberries [5, 11, 12] and blackberries [28] produced differing amounts of drip. The higher amounts of drip in riper fruit is also in agreement with studies that found that red blackberries produced less drip than black blackberries [28].

#### 3.1.3. Firmness

**Figure 3:**
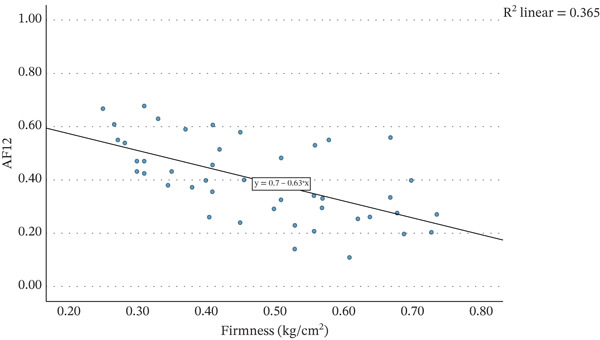
Scatter graph showing the mean firmness (kg/cm^2^) of strawberries plotted against the mean acceptance factor of nectars produced from these strawberries after 12 weeks of storage.

Firmness was significantly correlated with AF0 (*r* = −0.397, *p* = 0.006), AF12 (*r* = −0.604, *p* < 0.001) and D12 (*r* = 0.463, *p* = 0.001). Figure 3 shows the regression model between firmness and AF12. The regression was statistically significant (*R* ^2^ = 0.351, *F*(1, 45) = 25.898, *p* < 0.001) indicating that 35.1% of the variability in AF12 could be explained by firmness. The regression coefficient (*B* = −0.627, 95% CI [0.576, 0.821]) suggests that a decrease of 1 kg/cm^2^ in firmness corresponds to an increase in AF12 by 0.627. This supports previous work that found that strawberry firmness correlated strongly with colour stability [24]. The connection between firmness and AF12 was markedly lower than that with drip loss, suggesting that firmness is not as good at predicting colour stability than drip loss.

**Figure 4:**
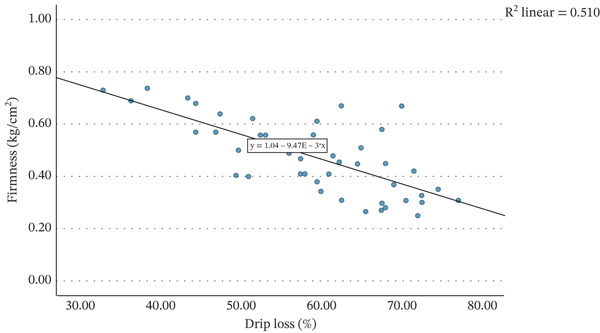
Scatter graph showing the firmness (kg/cm^2^) of strawberries plotted against the drip loss (%) of these strawberries after defrosting for 24 h at 20°C.

Figure 4 illustrates the relationship between strawberry firmness and percentage drip loss. A strong and significant negative correlation was observed (*r* = −0.714, *p* < 0.001), supporting findings from previous research that softer fruits exhibit higher drip loss percentages [4, 19]. Firmness is a recognised indicator of strawberry ripeness [20, 21] and correlates strongly with colour stability [24]. However, firmness measurements cannot be performed on frozen or thawed fruit, meaning this parameter is not suitable for assessing ripeness or predicting colour stability in frozen strawberries. As drip loss and firmness are highly correlated, one could be used as an indirect measure of the other, depending on if the raw material is frozen or fresh.

### 3.2. Solid Content Trials

#### 3.2.1. Drip Loss

The average drip loss of the strawberries used in these experiments was 69.9*%* ± 2.0*%*. Processing the solid fruit fraction (following the removal of the liquid drip loss) into puree involved sieving, which removed seeds and stalks, further decreasing the weight of the solid fraction. After sieving, the liquid fraction accounted for 73.3*%* ± 2.3*%* of the original fruit mass. Purees containing 25% solid content (where the solid content is defined as the percentage weight of the puree after processing and liquid removal) therefore most closely represent purees prepared under typical solid‐to‐liquid ratios. These purees can be considered broadly comparable to nectars produced without prior separation of the liquid and solid components.

#### 3.2.2. Colour Stability

Table 1 presents the mean AF0, AF12 and D12 values for nectars prepared from the reconstituted purees. Nectar colour was measured using reflectance, which is the necessary method to track degradation through AF of nectars made of puree [25]. Samples with no solid content (0%) lacked sufficient turbidity for reliable reflectance measurements, as AF could not be calculated from reflectance measurements of these samples. For these samples, colour was instead measured using transmission which allowed AF to be calculated, and the degradation could be tracked; however, it was impossible to directly numerically compare AF values from transmission to the AF values from reflectance, just the trends in degradation. Due to these differences, these data points were excluded from the statistical analysis. Significant differences (*p* < 0.05) in AF0 and AF12 were observed among nectars with varying proportions of solid content.

**Table 1:** The acceptance factor of nectar when initially produced (AF0), after 12 weeks of storage at 20°C (AF12), and the difference between these two points (D12), produced from purees made from different proportions of solid; where the solid is what is left after strawberries were defrosted for 24 h at room temperature and liquid drip collected separately from the strawberries.

Solid %	AF0	AF12	D12
0	1.406 ± 0.054	1.052 ± 0.060	0.352 ± 0.087
5	0.684 ± 0.045^f^	0.427 ± 0.046^f^	0.257 ± 0.055^a^
10	0.754 ± 0.019^e^	0.527 ± 0.071^e^	0.227 ± 0.079^a^
25	0.874 ± 0.020^d^	0.711 ± 0.032^d^	0.162 ± 0.016^b^
50	0.997 ± 0.025^c^	0.860 ± 0.052^c^	0.137 ± 0.032^bc^
75	1.050 ± 0.040^b^	0.960 ± 0.033^b^	0.090 ± 0.020^cd^
100	1.101 ± 0.015^a^	1.021 ± 0.034^a^	0.079 ± 0.022^d^

*Note:* Different lower case letters (vertical) illustrate significant differences (*p* < 0.05). Samples with 0% solid (liquid) were not included in ANOVA calculations of AF0, AF12 and D12 as these were measured with transmission rather than reflectance and so they have no letter.

**Figure 5:**
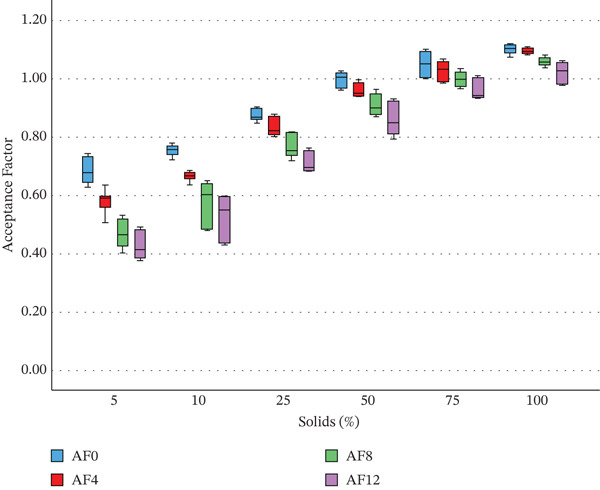
The acceptance factor on initial production (AF0), after 4 weeks (AF4), 8 weeks (AF8) and 12 weeks (AF12) of storage of nectars made from purees defined by the percentage of solids; where the solid is what is left after strawberries were defrosted for 24 h at room temperature and liquid drip collected separately from the strawberries.

From initial production, nectars with lower solid content were less acceptable to consumers compared with those with higher solid content, a trend that remained consistent after 12 weeks of nectar storage. Figure 5 illustrates this by showing the degradation of AF over time across the different solid content levels. Samples with higher solid content exhibited higher AF0 values and demonstrated a slower rate of decline, resulting in significantly higher AF12 values. These results are further supported by the strong correlations between solid content percentage and both AF0 and AF12 (*r* = 0.941 and 0.937, respectively; *p* < 0.001). Additionally, D12 showed a strong negative correlation with solid content percentage (*r* = −0.801; *p* < 0.001).

These findings suggest that nectars produced from purees with a higher proportion of solids (i.e., with less of the liquid released during defrosting) exhibit colour that is initially more acceptable to consumers (higher AF0) and display greater colour stability (indicated by a smaller D12). To enhance nectar colour stability, removing liquid drip to increase the proportion of solids is therefore recommended. Moreover, during puree processing, it is important to maximise yield during the sieving stage to retain as much solid material as is possible, as reduced solids would otherwise result in lower consumer acceptance and poorer colour stability.

This phenomenon may also explain occasional anomalies in colour stability (i.e., if a nectar is unexpectedly more stable or less stable). If defrosted strawberries have been used to produce nectars without careful attention to maintaining the correct proportions, then nectars could comprise an unnatural ratio of solids and liquids, potentially affecting their colour stability.

In commercial processing, intentionally excluding some of the liquid drip could be used to produce nectars with improved colour stability. However, this approach would lower overall product yields and could be economically unfeasible for industrial producers. To make this method more viable, the liquid drip could be diverted to alternative product streams—for example, sold as juice for concentrate—allowing producers to enhance nectar colour stability without sacrificing profitability.

#### 3.2.3. TSS and TA

Table 2 shows the TSS, TA and pH value of each of the different puree blends. TSS increased with increasing percentage of solids; this could explain the increase in colour acceptance and colour stability, as although the majority of the soluble solids are sugars [36], there are several minor compounds that are associated with nectar colour stability [37]. The most significant of which are anthocyanins, that will be discussed in Section 3.2.4. In addition, other polyphenol compounds such as *epi*afzelchin have been found to have a positive effect on nectar colour stability [37]. Other phenolics (including flavonoids, tannins and phenolic acids) and metal ions [38] can act as natural copigments [39], which contribute to nectar colour stability [40]. Higher proportions of these compounds, reflected in the higher TSS, could account for the greater stability at higher drip loss. TA values increased with percentage of solids in the puree blends, although there was no statistically significant difference in TA between the liquid drip (0%) and the solid fraction (100%) (*t*(4) = −2.2, *p* = 0.092). Despite this, a significant positive correlation was observed between TA and solid percentage (*r* = 0.614, *p* < 0.001). A lower pH value stabilises the red coloured flavylium cations [41], giving better, redder colour (more acceptable to consumers), but despite the increasingly higher TA content as the solid contents increased, the pH did not decrease in the same way, and there was no significant correlation between TA and pH (*r* = −0.116, *p* = 0.292), and so increased TA may not be the cause of the increased stability.

**Table 2:** Total soluble solids, pH value and titratable acidity of purees made from different proportions of solid; where the solid is defined as the fraction which is left after strawberries were defrosted for 24 h at room temperature and liquid drip collected separately from the strawberries.

Solid %	Total soluble solids (°Brix)	Titratable acidity (g/L)	pH value
0	6.2 ± 0.2^b^	9.3 ± 0.8^a^	3.47 ± 0.08^ab^
5	6.3 ± 0.3^b^	9.1 ± 0.7^a^	3.46 ± 0.05^ab^
10	6.3 ± 0.4^b^	9.3 ± 0.9^a^	3.54 ± 0.09^a^
25	6.5 ± 0.4^ab^	9.5 ± 0.8^a^	3.50 ± 0.11^a^
50	6.9 ± 0.6^ab^	10.1 ± 1.0^a^	3.36 ± 0.11^b^
75	7.0 ± 0.3^ab^	10.3 ± 0.8^a^	3.55 ± 0.02^a^
100	7.6 ± 0.5^a^	10.7 ± 0.9^a^	3.44 ± 0.18^ab^

*Note:* Different lower case letters (vertical) illustrate significant differences (*p* < 0.05).

#### 3.2.4. Anthocyanin Content

Table 3 presents the average concentrations of individual anthocyanins as well as the total anthocyanin content for each solid percentage. The relative proportions of the different anthocyanins remained consistent across the varying solid content percentages, suggesting that the removal of liquid to improve colour stability did not alter the anthocyanin profile of the nectars. This consistency is important, as the anthocyanin profile is known to influence colour stability [31]. The liquid fraction had a significantly lower total anthocyanin concentration than the solid fraction (*t*(16) = −7.2, *p* < 0.001). Although AF0 and AF12 values were statistically distinguishable between the different solid content levels, anthocyanin concentrations did not follow the same pattern. However, a significant difference was detected between nectars prepared from purees containing ≥ 50% solids and those with < 50% solids (*t*(61) = 8.9, *p* < 0.001).

**Table 3:** Concentration of different anthocyanins (mg/100 g fresh nectar weight) in nectars made from purees of different percentage of solid, where the solid is defined as the solid fraction left after drip loss separation—where strawberries were defrosted for 24 h at room temperature and liquid drip collected separately from the strawberries. Different lower case letters (vertical) illustrate statistical differences (*p* < 0.05).

Solid %	Cyanidin‐3‐o‐glucoside	Pelargonidin‐3‐o‐glucoside	Pelargonidin‐3‐o‐rutinoside	Pelagonidin‐3‐o‐(6 malonyl glucoside)	Pelargonidin‐3‐succinyl arabinose	Total anthocyanin content
0	0.07 ± 0.05^d^	10.07 ± 2.67^c^	0.58 ± 0.15^c^	1.55 ± 0.44^c^	0.08 ± 0.02^cd^	12.35 ± 3.30^c^
5	0.08 ± 0.06^d^	10.50 ± 3.13^c^	0.57 ± 0.15^c^	1.63 ± 0.52^c^	0.06 ± 0.02^d^	12.85 ± 3.89^c^
10	0.10 ± 0.08^cd^	11.24 ± 2.78^c^	0.59 ± 0.12^c^	1.84 ± 0.50^c^	0.08 ± 0.02^cd^	13.89 ± 3.48^c^
25	0.20 ± 0.06^bc^	13.97 ± 3.18^bc^	0.66 ± 0.13^bc^	2.33 ± 0.60^bc^	0.10 ± 0.02^bc^	17.25 ± 3.98^bc^
50	0.27 ± 0.04^b^	15.91 ± 2.50^b^	0.64 ± 0.11^bc^	2.95 ± 0.52^b^	0.13 ± 0.01^ab^	19.89 ± 3.17^b^
75	0.44 ± 0.11^a^	22.42 ± 3.96^a^	0.91 ± 0.17^ab^	4.263 ± 0.978^a^	0.16 ± 0.03^a^	28.19 ± 5.18^a^
100	0.45 ± 0.09^a^	21.43 ± 4.00^a^	0.85 ± 0.20^a^	4.081 ± 0.886^a^	0.14 ± 0.03^a^	26.95 ± 5.10^a^

*Note:* Lower case letters (horizontal) indicates significant difference (*p* < 0.05).

Notably, all samples containing 75% solids showed higher anthocyanin concentrations than those with 100% solids, although this difference was not statistically significant. One possible explanation is that the 100% solids samples were stored as a puree for a longer period prior to nectar production and pasteurisation. The extended storage may have caused some anthocyanin degradation prior, reducing anthocyanin content in the final product, as anthocyanins in strawberry products are known to degrade during storage [26]. This is due to during thawing, and particularly after pureeing, cell breakdown allows the enzymes that degrade anthocyanins (PPO/POD) to come into contact with phenolics, which initiates anthocyanin breakdown. The fact that the anthocyanins in 75% was higher, but the AF0, AF12 and D12 was higher in the 100% further confirms previous findings that anthocyanin concentration is not the only factor to impact colour stability [27, 41]. In this case, keeping the purees at 4°C could have led to greater copigmentation of phenolics, which impacts colour stability [39, 40].

Total anthocyanin content was strongly correlated with the percentage of solid content in puree used for the nectars (*r* = 0.818, *p* < 0.001), as well as with AF0, AF12 and D12 (*r* = 0.839, 0.846 and −0.741, respectively, *p* < 0.001). These results corroborate previous findings indicating both colour acceptance and colour stability are highly influenced by anthocyanin content, with higher content in more stable strawberry products [15, 21, 23]. However, the observation that the nectars with 75% solids had a higher anthocyanin concentration than those with 100% solids, yet the latter still achieved superior AF0, AF12 and D12 outcomes, indicates that anthocyanin concentration alone is not the sole factor defining colour stability or consumer acceptance. Additionally, although the anthocyanin content is very important for the colour, it is not the sole determinant of colour stability [27, 41], as colour stability can be affected by copigmentation, as mentioned in Section 3.2.3. The colour stability of strawberry nectars is also defined by the enzyme activity. Although enzymes are dislocated during the thawing, it is possible that these enzymes are removed with the liquid drip. Studies in peach and kiwi found that the freezing affected both the drip loss and the enzyme activities, which could account for the differences observed [42, 43].

#### 3.2.5. Sugar and Acid Content

##### 3.2.5.1. Sugar Content

The mean TSS (expressed in °Brix) for each of the different puree blends is summarised in Table 2, whereas the mean concentrations of different sugars analysed by HPLC‐RID in the liquid fraction (0%) and solid fraction (100%) are summarised in Table 4. A significant difference in TSS was observed between the liquid fraction (0%) and the solid fraction (100%) (*t*(4) = −4.53, *p* = 0.011). As discussed in Section 3.2.3, the TSS of the purees increased with increasing percentage of solid content, but this trend was not reflected in the sugar concentrations analysed by HPLC‐RID. Here, the solid portion had a significantly lower concentration of total sugars compared with the liquid fraction (*t*(1.6) = 3.74, *p* = 0.002). Previous studies have shown that sugar content (as analysed by HPLC‐RID) correlates with TSS [44], although other studies found no correlation between the amount of individual sugars and TSS [45]. The discrepancy between the TSS and the total sugars observed in this study shows that there are components in the TSS beyond the tested sugars that contribute to the TSS, as discussed in Section 3.2.3. For the liquid fraction to be suitable for direct juice sale, recommended thresholds include a minimum TSS of 6.3°Brix, glucose content between 15–35 g/L, fructose between 18–40 g/L, sucrose levels < 10 g/L, and a glucose: fructose ratio between 0.75 and 1 [46]. In this study, the average TSS in the liquid portion was slightly below this recommended threshold, although TSS values were observed across all samples produced in these trials. Similarly, both glucose and fructose fell below the suggested ranges, although the glucose: fructose ratio remained within the acceptable range at 0.90. Sucrose levels exceeded the recommended minimum, but sucrose was consistently high in all samples, likely reflecting the naturally high sucrose content of the strawberry raw material used. As direct strawberry juice is a relatively rare product, the liquid drip would be more realistically processed into concentrated juice. In this case, the lower sugar concentrations would not pose a limitation, as they could be adjusted during concentration, making this a viable processing option for the discarded liquid fraction.

**Table 4:** The mean and standard deviation of different sugars (mg/g fresh puree weight) as determined by HPLC‐RID, in the liquid drip (0% solid) and the processed solids (100%).

Sugar	0% solid (liquid fraction)	100% solid (solid fraction)
Sucrose	14.3 ± 1.4^a^	13.2 ± 0.7^b^
Glucose	14.1 ± 1.1^b^	12.4 ± 1.2^a^
Fructose	15.6 ± 0.6^a^	13.6 ± 0.9^b^
Total Sugars	44.0 ± 3.1^a^	39.2 ± 2.3^b^

*Note:* Lower case letters (horizontal) indicates significant difference (*p* < 0.05).

##### 3.2.5.2. Acid Content

The mean TA (expressed in g/L) for each of the different puree blends is summarised in Table 2, whereas Table 5 summarises the mean concentrations of individual acids, determined via HPLC‐UV, in the liquid fraction (0%) and the solid fraction (100%). Ascorbic acid content was significantly higher in the liquid fraction than the solid fraction. Since ascorbic acid is known to degrade anthocyanin pigments [47], and high concentrations have been reported to correlate negatively with colour stability [48, 49], this may partially explain why the liquid fraction, and thus nectars with higher percentages of liquid, had poorer colour stability. Although the liquid fraction contained higher ascorbic acid concentrations, the total acid content, as analysed by HPLC‐UV, did not differ significantly between the solid and liquid fractions (*t*(1.4) = −0.56, *p* = 0.293). Professional guidelines recommend that juice should have a TA content of 5.1–11.5 g/L [31], and all the purees produced in this study, regardless of solid content, met this criterion. Additionally, the citric acid content, which is recommended to fall within the range of 5–11 g/L [31] exceeded this range in both the liquid and solid fractions. Therefore, it appears that the raw material used in these trials was inherently unsuitable for NFC (not from concentrate) juice production, rather than being a consequence of the separation of liquid and solid.

**Table 5:** The mean and standard deviation of different acids as determined by HPLC‐UV (mg/g fresh puree weight) in the liquid drip (0% solid) and the processed solids (100%).

Acid	0% solid (liquid fraction)	100% solid (solid fraction)
Ascorbic acid	0.09 ± 0.05^a^	0.01 ± 0.00^b^
Citric acid	11.38 ± 1.78^a^	11.32 ± 1.29^a^
Malic acid	2.49 ± 0.19^b^	3.00 ± 0.20^a^
Shikimic acid	0.04 ± 0.001^a^	0.04 ± 0.00^a^
Fumaric acid	0.02 ± 0.00^a^	0.02 ± 0.00^a^
Total acids	13.92 ± 2.00^a^	14.39 ± 1.49^a^

*Note:* Lower case letters (horizontal) indicates significant difference (*p* < 0.05).

The lack of significant differences between the liquid and solid fractions indicates that removing liquids to improve nectar stability is unlikely to adversely affect the acid content of the final products. Juice produced solely from the liquid fraction would likely be similar to juice made from the whole fruit. The sugar: acid ratio of the liquid fraction was 6.8, which is lower than typical values. However, the ratio for the 25% blend (which approximates the ratio expected from standard, nonseparated raw material) was also 6.8 and only marginally higher at 7.3 in the solid portion. This demonstrates that although separating drip loss slightly altered the sugar–acid ratio, these differences were not substantial. The relatively low ratio observed in this study primarily reflects the naturally low sugar content of the strawberries used in these trials.

## 4. Conclusions

The results of this study yield two important findings: Firstly, strawberry drip loss serves as an approximate indicator of the colour stability of nectars produced from these strawberries. Specifically, strawberries with higher levels of drip loss result in nectars with superior colour stability. This insight is particularly valuable for small industrial producers, as measuring drip loss is a straightforward procedure that does not require specialist equipment or training. Moreover, defrosted strawberries with high percentages of drip loss are generally unsuitable for products where texture and appearance are critical. Therefore, by quantifying drip loss, producers can use drip loss as a screening tool to determine optimal product allocation: Strawberries with high drip percentages should be prioritised for nectar production, whereas those with lower drip loss can be used for applications where maintaining texture and appearance is essential.

Secondly, separating the liquid and solid fractions during the thawing process and increasing the proportion of the solid fraction significantly enhanced the initial colour, colour after storage and colour stability of the resulting nectar. This study is the first to demonstrate that removing the liquid drip from defrosted strawberries during strawberry nectar production offers a practical and effective strategy to improve the colour stability of strawberry nectars; however, removing drip loss would decrease yields and is therefore an economically disadvantageous method. In addition, the composition of liquid drip differed significantly from that of the solid fraction. Despite these limitations, the liquid fraction remained suitable for alternative processing streams, such as in production of juice concentrate.

Future work should repeat the drip loss removal method for other cultivars of strawberries, especially cultivars popular in areas where small scale nectar production is popular, as different cultivars are known to respond differently to different processing. Especially as different cultivars were shown to have very different quantities of drip loss, which would impact how much liquid drip could be removed. Furthermore, more investigations could be performed on the differences between the liquid and solid fractions to determine factors such as enzyme activity and other polyphenols, which could impact the colour stability.

**Nomenclature**
AF: Acceptance Factor
AF0: Acceptance Factor on day of production
AF4: Acceptance Factor after 4 weeks
AF8: Acceptance Factor after 8 weeks
AF12: Acceptance Factor after 12 weeks
D4: difference in Acceptance Factor between Week 0 and Week 4
D8: difference in Acceptance Factor between Week 0 and Week 8
D12: difference in Acceptance Factor between Week 0 and Week 12
TSS: total soluble solids
TA: titratable acidity

## Funding

This study was supported by the H2020 Marie Skłodowska‐Curie Actions (10.13039/100010665) (956257).

## Conflicts of Interest

The authors declare no conflicts of interest.

## Data Availability

The data that support the findings of this study are available from the corresponding author upon reasonable request.
